# A Simplified Method to Measure Choroidal Thickness Using Adaptive Compensation in Enhanced Depth Imaging Optical Coherence Tomography

**DOI:** 10.1371/journal.pone.0096661

**Published:** 2014-05-05

**Authors:** Preeti Gupta, Elizabeth Sidhartha, Michael J. A. Girard, Jean Martial Mari, Tien-Yin Wong, Ching-Yu Cheng

**Affiliations:** 1 Singapore Eye Research Institute and Singapore National Eye Centre, Singapore, Singapore; 2 Department of Ophthalmology, Yong Loo Lin School of Medicine, National University of Singapore and National University Health System, Singapore, Singapore; 3 Department of Biomedical Engineering, Faculty of Engineering, National University of Singapore, Singapore; 4 Department of Bioengineering, Faculty of Engineering Science, University College London, London, United Kingdom; 5 Saw Swee Hock School of Public Health, National University of Singapore and National University Health System, Singapore, Singapore; 6 Centre for Quantitative Medicine, Office of Clinical Sciences, Duke-NUS Graduate Medical School, Singapore, Singapore; Zhongshan Ophthalmic Center, China

## Abstract

**Purpose:**

To evaluate a simplified method to measure choroidal thickness (CT) using commercially available enhanced depth imaging (EDI) spectral domain optical coherence tomography (SD-OCT).

**Methods:**

We measured CT in 31 subjects without ocular diseases using Spectralis EDI SD-OCT. The choroid-scleral interface of the acquired images was first enhanced using a post-processing compensation algorithm. The enhanced images were then analysed using Photoshop. Two graders independently graded the images to assess inter-grader reliability. One grader re-graded the images after 2 weeks to determine intra-grader reliability. Statistical analysis was performed using intra-class correlation coefficient (ICC) and Bland-Altman plot analyses.

**Results:**

Using adaptive compensation both the intra-grader reliability (ICC: 0.95 to 0.97) and inter-grader reliability (ICC: 0.93 to 0.97) were perfect for all five locations of CT. However, with the conventional technique of manual CT measurements using built-in callipers provided with the Heidelberg explorer software, the intra- (ICC: 0.87 to 0.94) and inter-grader reliability (ICC: 0.90 to 0.93) for all the measured locations is lower. Using adaptive compensation, the mean differences (95% limits of agreement) for intra- and inter-grader sub-foveal CT measurements were −1.3 (−3.33 to 30.8) µm and −1.2 (−36.6 to 34.2) µm, respectively.

**Conclusions:**

The measurement of CT obtained from EDI SD-OCT using our simplified method was highly reliable and efficient. Our method is an easy and practical approach to improve the quality of choroidal images and the precision of CT measurement.

## Introduction

The choroid is important to support retinal and visual function as it supplies nutrients and oxygen to retinal pigment epithelial (RPE) cells and photoreceptors [1]. Therefore, the choroid may play a role in the pathophysiology of many vision threatening retinal diseases such as age-related macular degeneration [2], [3], polypoidal choroidal vasculopathy [4], [5], central serous chorioretinopathy [6], [7], Vogt-Koyanagi-Harada [8] disease and myopic macular degeneration [9]–[12]. To elucidate the mechanisms through which the choroid affects these retinal diseases, quantitative assessment of choroidal characteristics such as choroidal thickness (CT) is required.

However, it was not trivial to image CT because of its posterior location and pigments in the RPE layer [13], until the advent of spectral domain optical coherence tomography (SD-OCT) with enhanced depth imaging (EDI). EDI SD-OCT has provided many new insights into choroidal qualitative morphology. However, to date a notable disparity exists between the CT measurements obtained in different studies. Such variations could be due to unavailability of a standardized and simple to use measurement method and absence of built-in automated software to measure CT in most of the commercially available OCT machines [14]. At present, most of the studies perform the measurements manually by using the in-built caliper system provided by the machine, which is prone to measurement errors. The manual method is also time- and effort-consuming, making it unfeasible especially when dealing with large population data.

As a result, while many studies have published the distribution of CT in patients with retinal diseases and normal controls [15]–[19], none of these studies have clearly described the method used in detailed to measure CT. Therefore, the reliability of the CT measurement methods in most of the papers is unknown. In this paper, we describe a simple, semi-automated, time-efficient method to measure CT using images acquired by EDI SD-OCT and available compensation algorithms. We aimed to assess the reliability of this new CT measurement technique in a sample of healthy eyes.

## Materials and Methods

### Study Subjects and Design

Data for this analysis were derived from the Singapore Malay Eye Study (SiMES), a population-based cross-sectional study of eye diseases in Malay adults, age ranged from 40–80 years living in Singapore. Details of the study design, sampling plan, and methodology have been reported elsewhere [20]. In brief, participants recruited in the current study underwent standardized and detailed ophthalmic examination, including Spectralis EDI SD-OCT imaging (see next section). The study was approved by the Institutional Review Board of Singapore Eye Research Institute. It followed the tenants of the Declaration of Helsinki and written informed consent was obtained from the subjects after explanation of the nature and possible consequences of the study.

Spectralis images of 31 subjects were randomly selected using a random number generated in Stata (College par, Texas, USA). Choroidal images were only selected from right eye of each subject as our measurement technique can be applied uniformly between right and left eyes. Therefore only the right eyes of the subjects were evaluated. In addition, selecting only one eye from individuals would help to avoid inter-eye correlation issue in statistical analysis. “Normal fundus” was defined as free of any macular or vitroretinal diseases on the basis of clinical fundus examination by experienced Ophthalmologist and the results of OCT imaging. Exclusion criteria for the normal participants included: best corrected LogMAR VA >0.3, evidence of macular or vitroretinal diseases, previous retinal or refractive surgery, past history of intraocular surgery, or clinical features compatible with a diagnosis of glaucoma suspect or glaucoma.

To evaluate the intra-grader and inter-grader reliability, 31 Spectralis images were randomly selected for the initial grading phase. Grader A and grader B, masked to subject characteristics and clinical diagnosis, independently graded these images to assess inter-grader reliability. In addition, grader A repeated the measurements after 2 weeks to assess intra-grader reliability. Both graders assessed the same sets of training images before commencing the grading task.

### EDI SD-OCT Imaging

CT was obtained using Spectralis SD-OCT with EDI modality (Wavelength: 870 nm; Heidelberg Engineering, Heidelberg, Germany) after pupil dilation using tropicamide 1% and phenylephrine hydrochloride 2.5%. Subjects’ keratometry readings and the refraction data were entered into the software program to estimate optical magnification and, therefore, to allow for more accurate comparisons across individuals. A single experienced examiner masked to the clinical diagnosis of the subject performed the EDI-OCT examination. Seven sections, each comprising 100 averaged scans (using the automatic averaging and eye tracking features of the proprietary device), were obtained in an angle of 5°–30° rectangle centered at the fovea. The horizontal section passing through the center of the fovea was selected for analysis. For each subject only the right eye was chosen for subsequent analysis.

### Measurement of Choroidal Thickness

The two major steps in our CT semi-automatic measurement protocol are: (A) post processing of images by adaptive compensation technique and (B) quantitative measurement of CT using Photoshop software. On average, measurement of CT at multiple locations (1.5 and 3 mm nasal and temporal to the fovea) requires approximately 1 minute per image.

#### (A) Adaptive compensation technique

**Figure 1:**
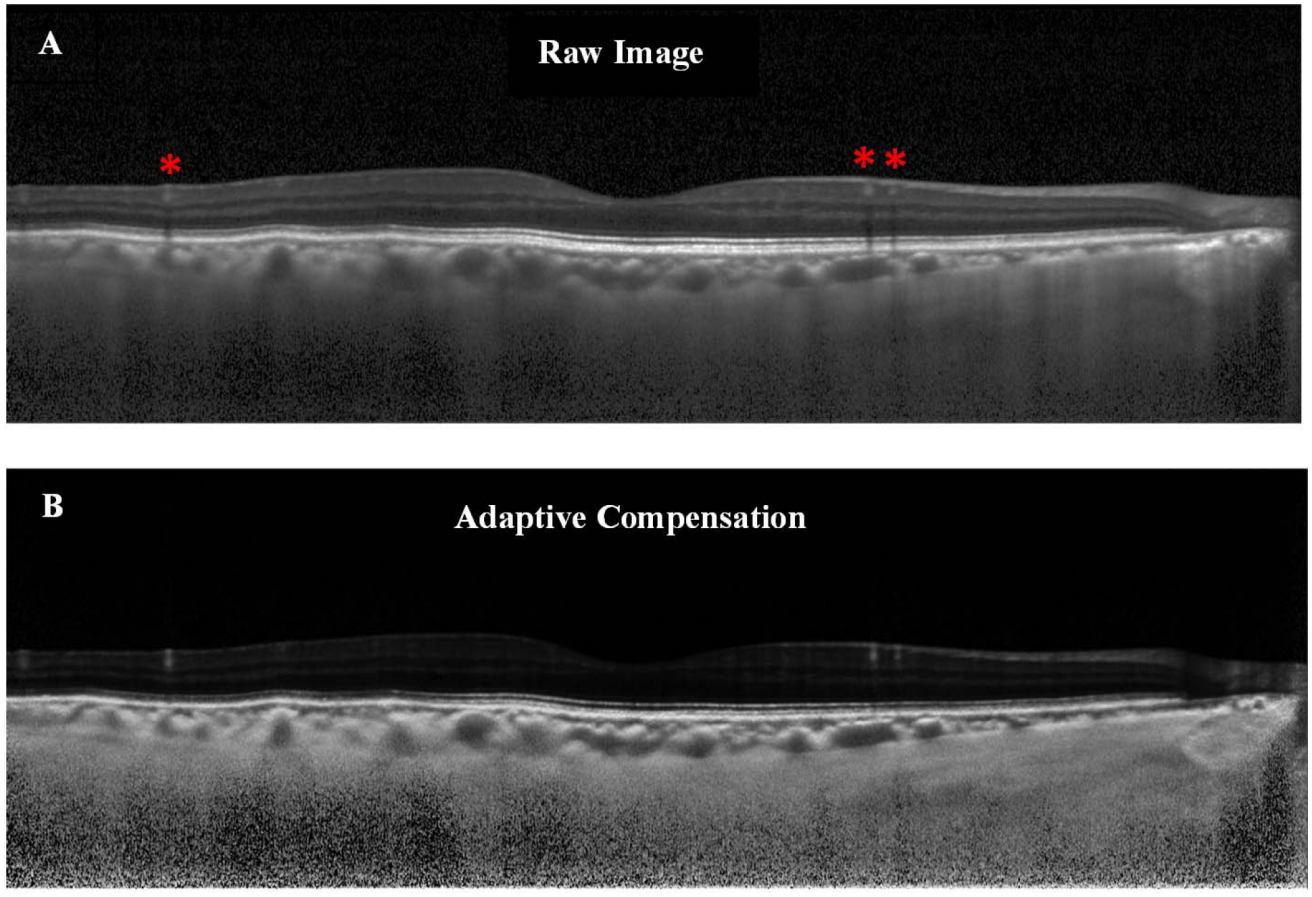
Illustration of the raw versus the compensated image. (A) Raw OCT image of a healthy subject. The choroid-scleral interface is only partially visible. Note the presence of blood vessels shadows as indicated by red asterisks. (B) Adaptive compensation was applied to the raw image in order to remove blood vessels shadows, enhance contrast and improve visibility of the choroid-scleral interface (more uniform).

An accurate evaluation of the CT with EDI-OCT mainly relies on how well one can delineate the choroid-scleral interface (CSI), which anatomically represents the junction between the choroid and the sclera, and is a principal landmark for quantitative measurements of choroid. However, at present identification of CSI is highly variable as there is no algorithm available in Spectralis for its automatic detection. Therefore, in order to accurately determine CT, once the EDI-OCT image was obtained, the CSI was enhanced using a novel post-processing compensation algorithm [21] which greatly improved the detection of CSI by correcting the deleterious effects of light attenuation. In brief, this novel adaptive compensation algorithm improved the ability to detect and visualize the CSI. First, it removes noise over-amplification at high depth and shadow artifacts casted by blood vessels (thus decreasing the intra-layer contrast of the choroid). Second, it improves the visibility of posterior choroid boundary, by significantly increasing the inter-layer contrast across the CSI (**Figure 1**).

#### (B) Measurement of CT using photoshop software

**Figure 2:**
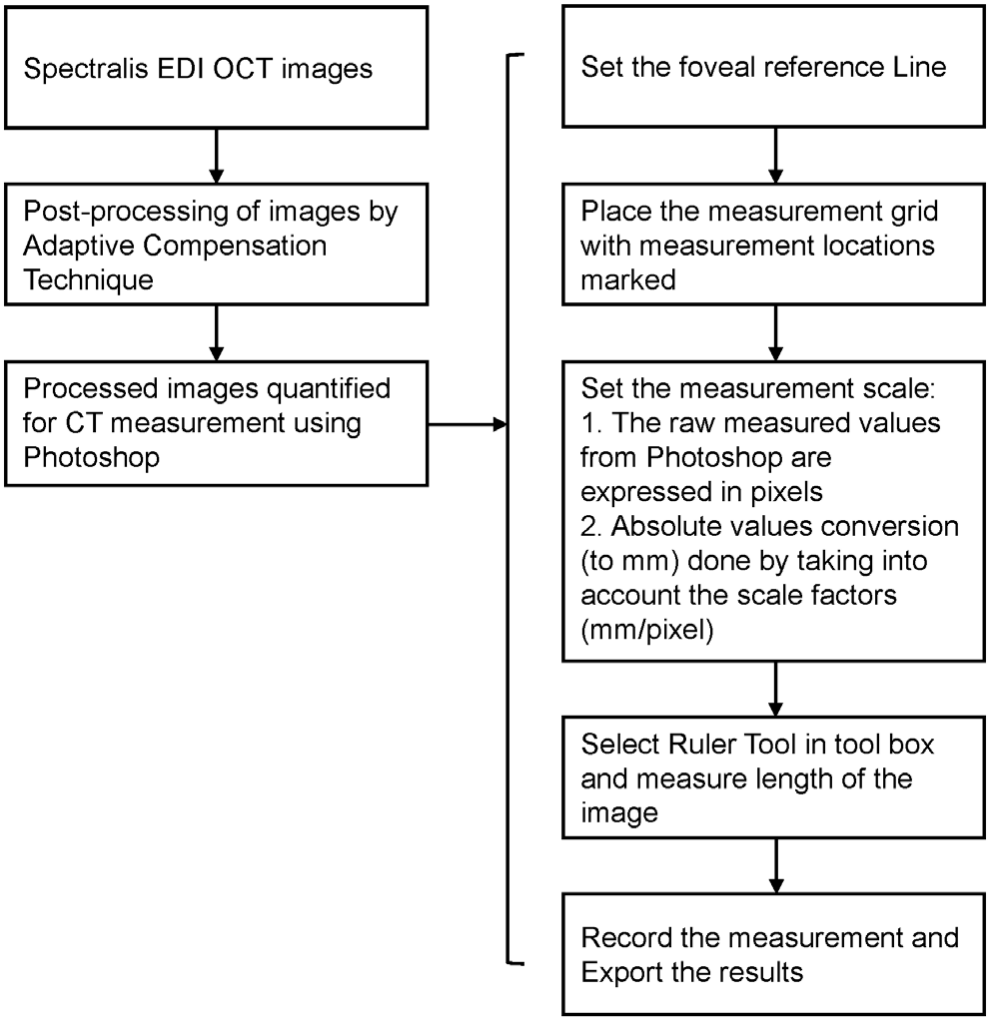
Flow diagram of the measurement protocol.

The enhanced images were measured in Photoshop CS6 extended (Adobe Systems Incorporated, San Jose, California). CT at sub-foveal, 1.5 mm and 3 mm nasal and temporal from the fovea were measured. **Figure 2** shows a flow diagram summarizing the CT measurement protocol. The detailed explanation of the steps involved in quantification of CT measurement using Photoshop is provided in Appendix S1.

### Statistical Analysis

Statistical analysis was performed using MedCalc Version 12.6 (MedCalc Software, Ostend, Belgium) and SPSS Version 20.0 (SPSS, Inc., Chicago, IL, USA). Shapiro-Wilk tests were used to check the normality of CT data at various locations. The reliability of the CT measurement was assessed using intra- and inter-grader agreements between two independent graders, measured by the absolute agreement model of the intra-class correlation coefficient (ICC) [22]. ICC value of 0.81–1.00 indicates almost perfect agreement. Values of less than 0.40 indicate poor to fair agreement. Bland Altman plot analyses [23], [24] were performed to see if there is any proportional bias between measurements. A two-tailed paired sample *t*-test was used to analyze differences between means in CT by location.

## Results

Images from 31 eyes of 31 participants (aged 64.4±7.4 years) were included in the analysis (**Table 1**). The mean (standard deviation) sub-foveal CT in this study was 230.37 (66.66) µm (average of 1^st^ and 2^nd^ measurements of grader A). CT measured by the two graders is summarized in **Table 2**. The mean sub-foveal CT measured by grader A for the 1st and 2^nd^ measurements was 229.74 (65.12) µm and 231 (69.14) µm, respectively, and was 232.19 (67.89) µm by grader B.

**Table 1:** Baseline characteristics of study subjects.

Characteristics	Mean (SD)
Age, years	64.35 (7.42)
Gender, male	11 (35.48)
Axial length, mm	23.33 (0.88)
Average choroidal thickness,* µm	
Sub-foveal	230.37 (66.66)
Nasal, 1.5 mm	214.33 (74.34)
Nasal, 3 mm	185.77 (69.96)
Temporal, 1.5 mm	224.53 (57.27)
Temporal, 3 mm	229.74 (52.03)

Data are expressed as mean (SD) except for gender, which is expressed as number (%).

*The average of 1st and 2nd measurement of Grader A.

**Table 2:** Summary of choroidal thickness measurements at various locations.

Location	Grader A, 1^st^ Measurement	Grader A, 2^nd^ Measurement	Grader B Measurement
Sub-foveal	229.74 (65.12)	231.00(69.14)	232.19 (67.89)
Nasal, 1.5 mm	213.84 (75.12)	214.84(75.49)	221.52(76.08)
Nasal, 3 mm	191.13 (72.79)	180.42(68.25)	187.55(71.06)
Temporal, 1.5 mm	223.94 (56.33)	225.13(59.24)	231.65 (60.82)
Temporal, 3 mm	228.68 (50.64)	230.81(54.57)	230.71 (53.80)

Data are mean (SD).

**Figure 3:**
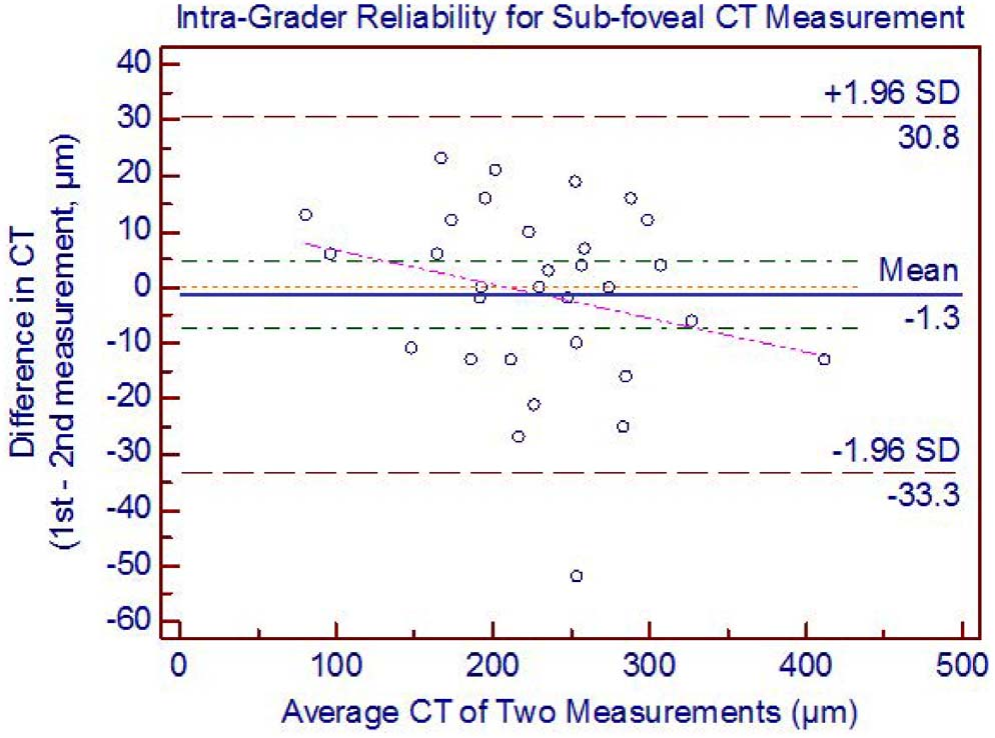
Bland Altman plot of intra-grader reliability of sub-foveal choroidal thickness (CT) measurement. The difference was calculated by the 1st measurement minus the 2nd measurement. Pink dashed line represents regression line of difference between 1st and 2nd measurements.

**Figure 4:**
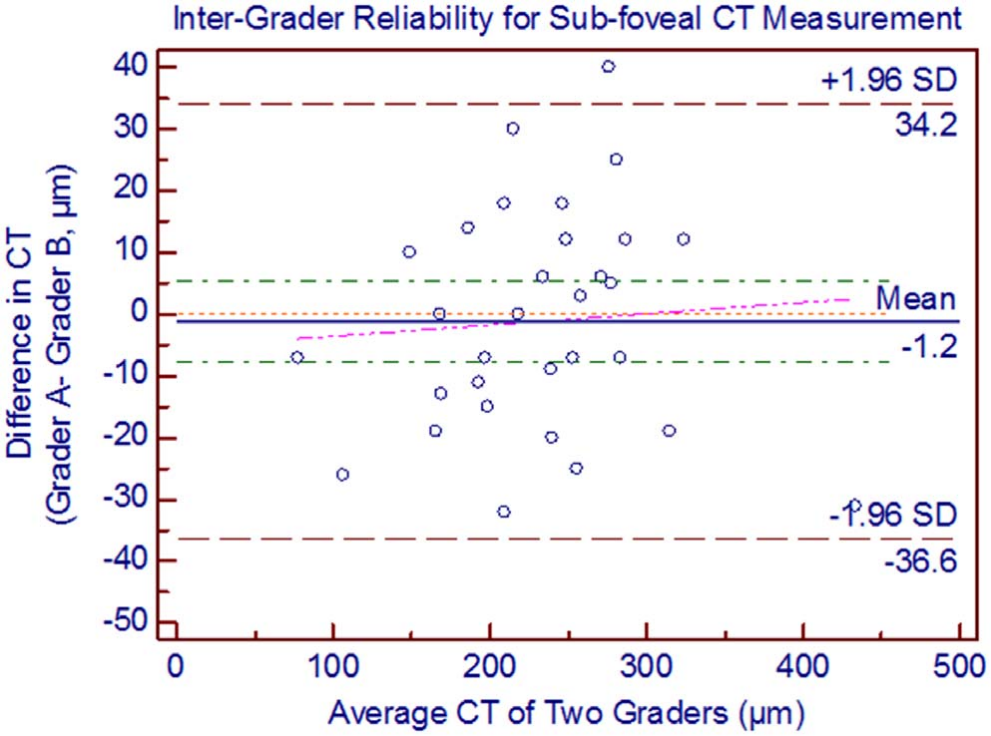
Bland Altman plots of inter-grader reliability of sub-foveal choroidal thickness (CT) measurement. The difference was calculated by the grader A measurement minus the grader B measurement. Pink dashed line represents regression line of difference between the two graders measurements.

Using adaptive compensation both the intra-grader reliability (ICC: 0.95 to 0.97) and inter-grader reliability (ICC: 0.93 to 0.97) were perfect for all five locations of CT (**Table 3**). However, with the conventional technique of manual CT measurements using built-in callipers provided with the Heidelberg explorer software, the intra- (ICC: 0.87 to 0.94) and inter-grader reliability (ICC: 0.90 to 0.93) for all the measured locations is lower (**Table 4**). Using adaptive compensation, the Bland Altman analysis of intra-grader reliability for sub-foveal CT measurement showed 95% LOA of −33.3 to 30.8 with a mean difference of −1.3 µm (**Figure 3**). No significant systemic (except at nasal 3 mm, p = 0.003) and proportional bias was detected in intra-grader CT measurements at all locations. The Bland Altman analysis of inter-grader reliability for sub-foveal CT measurement showed 95% LOA of −36.6 to 34.2 with a mean difference of −1.2 µm (**Figure 4**). No significant proportional bias was observed in the inter-grader CT measurements at all locations. Nonetheless, a significant systemic bias at both 1.5 and 3 mm nasal locations was found in the inter-grader CT measurement comparison (p = 0.001). However, this could be due to thinnest CT at nasal locations, making it more prone to systemic bias in CT measurements.

**Table 3:** Intra- and inter-grader agreements for the choroidal thickness measurement at 5 horizontal locations using adaptive compensation.

					Assessment of proportional bias^d^	
	Locations, CT measurement	ICC (95% CI)	Mean difference(95% LOA)	P Value^c^	Pearson’s correlation coefficient, r	P Value
Intra-graderReliability^a^	Sub-foveal	0.97 (0.94 to 0.98)	−1.3 (−33.3 to 30.8)	0.672	0.248	0.179
	Nasal, 1.5 mm	0.95 (0.90 to 0.97)	−1.0 (−48.0 to 46.0)	0.818	−0.160	0.933
	Nasal, 3 mm	0.95 (0.87 to 0.98)	10.7 (−25.3 to 46.7)	0.003	0.249	0.176
	Temporal, 1.5 mm	0.96 (0.92 to 0.98)	−1.2 (−31.9 to 29.5)	0.674	−0.187	0.313
	Temporal, 3 mm	0.95 (0.90 to 0.97)	−2.1 (−33.5 to 29.2)	0.464	−0.248	0.178
Inter-graderReliability^b^	Sub-foveal	0.96 (0.93 to 0.98)	−1.2 (−36.6 to 34.2)	0.715	0.070	0.708
	Nasal, 1.5 mm	0.97 (0.92 to 0.99)	−9.0 (−34.4 to 16.4)	0.001	−0.127	0.495
	Nasal, 3 mm	0.97 (0.89 to 0.98)	−9.8 (−37.4 to 17.8)	0.001	−0.230	0.212
	Temporal, 1.5 mm	0.93 (0.86 to 0.96)	−6.5 (−48.3 to 35.3)	0.099	−0.075	0.687
	Temporal, 3 mm	0.94 (0.88 to 0.97)	0.1 (−35.5 to 35.7)	0.977	0.043	0.818

LOA, Limits of Agreement; ICC, Intraclass Correlation Coefficient; CI, Confidence Interval.

a Mean difference was determined from the1^st^ time measurement minus the 2^nd^ time measurement.

b Mean difference was determined from Grader A measurement minus Grader B measurement.

c P value of one sample t-tests (comparing between mean difference and zero value) to indicate presence of systemic bias.

d Using Pearson’s correlation coefficients of regression line.

**Table 4:** Intra- and inter-grader agreements for the choroidal thickness measurement at 5 horizontal locations using Spectralis SD-OCT with conventional manual technique.

	Locations, CT measurement	ICC (95% CI)	Mean difference (95% LOA)
Intra-grader Reliability^a^	Sub-foveal	0.94 (0.87to 0.97)	9.7 (−30.3 to 49.7)
	Nasal, 1.5 mm	0.92 (0.85 to 0.96)	2.9 (−41.1 to 54.9)
	Nasal, 3 mm	0.92 (0.85 to 0.96)	4.1 (−36.1 to 44.3)
	Temporal, 1.5 mm	0.87 (0.75 to 0.93)	−7.0 (−52.1 to 38.1)
	Temporal, 3 mm	0.88 (0.77 to 0.94)	−1.9 (−44.9 to 48.6)
Inter-grader Reliability^b^	Sub-foveal	0.90 (0.60 to 0.96)	−19.1 (−63.7 to 25.6)
	Nasal, 1.5 mm	0.93 (0.68 to 0.97)	18.3 (−59.7 to 23.2)
	Nasal, 3 mm	0.91 (0.67 to 0.96)	−15 (−54.3 to 24.3)
	Temporal, 1.5 mm	0.91 (0.72 to 0.96)	−12.7 (−49.8 to 24.4)
	Temporal, 3 mm	0.90 (0.58 to 0.96)	−15.7 (−51.3 to 19.9)

LOA, Limits of Agreement; ICC, Intraclass Correlation Coefficient; CI, Confidence Interval.

a Mean difference was determined from the1^st^ time measurement minus the 2^nd^ time measurement.

b Mean difference was determined from Grader A measurement minus Grader B measurement.

In addition, the choroid intra-layer contrast (a measure of shadow presence when high) and the CSI inter-layer contrast (a measure of boundary visibility when high) were computed for all images (n = 31) before and after applying adaptive compensation (as in [25]). We found that the intra-layer contrast significantly decreased from 0.84±0.07 to 0.60±0.07 (p<0.001; *t*-test), whereas the inter-layer contrast significantly increased from 0.50±0.14 to 0.90±0.10 (p<0.001; paired *t*-test) after applying adaptive compensation (**Table 5**).

**Table 5:** Intra- and inter-layer contrast (n = 31 subjects).

		Mean (SD)	P-Value
Intra-layer Contrast	Standard (without compensation)	0.84 (0.07)	<0.001
	Adaptive Compensation	0.60 (0.07)	
Inter-layer Contrast	Standard (without compensation)	0.50 (0.14)	<0.001
	Adaptive Compensation	0.90 (0.10)	

## Discussion

Despite significant advances in imaging technology there are considerable variations in CT measurements across clinical studies. In this report, we described a simple, semi-automated method using adaptive compensation to measure CT from images acquired by EDI SD-OCT. The results showed that the method permits a highly reproducible tool for assessing CT from EDI SD-OCT images. Our measurement method is also simple and requires little time to perform (on average ∼1 minute per image). With these features, our method may have great potential for use in both clinical and population-based studies which involve large number of images.

There are considerable differences in CT measurements across studies. Even though advances in OCT technology have reduced acquisition time, adequate visualization of choroid is still lacking. There are several challenges in imaging the choroid. First, the choroid is located behind the RPE and Bruch’s membrane, making it less accessible than the retina and more difficult to be visualized. Second, the pigmentation in the RPE and the choroid itself decreases the signal intensity. Lastly, unlike retinal imaging [26], wherein the RPE and internal limiting membrane are thin and clearly identifiable, the transition zone between the choroid and sclera (i.e. CSI) has blurred border and is broader, making CSI difficult to identify.

However, it is not always easy to distinguish CSI on acquired images, which may be related to scan quality or anatomic variation. Some choroidal scans have a distinct hypo-reflective line corresponding to the supra-choroidal space, but often this line can be indistinct leading to measurement error [27]. At present, identification of CSI is highly variable. To overcome these challenges, in this study we enhanced choroidal-scleral junction using a post-processing adaptive compensation algorithm [21]. Measurement of CT was performed in the post-processed images using Photoshop (Adobe Systems Incorporated, San Jose, California), a readily available and easy-to-use program.

Adaptive compensation provides two major improvements. First, the intra-layer contrast (of the choroid) significantly decreased after applying adaptive compensation, indicating successful shadow correction within the choroid. Second, the inter-layer contrast (across the CSI) significantly increased after applying adaptive compensation, indicating better visualization of CSI and therefore allowing for precise measurement of CT. These results are consistent with a previous study on standard compensation [25] and indicate significant improvements in image quality. Also, a study by Lin *et al.* established outer choroidal contrast as a valid quantifiable measure of choroidal image quality and demonstrated that inverted SD-OCT imaging optimizes visualization of CSI and choroidal vessels through improved outer choroid contrast [28].

With improved intra- and inter-layer contrast, the compensated images are likely more accurate than the non-compensated images as the deleterious effects of OCT light attenuation have been corrected, thus making the CSI more visible. The post-compensation images are more representative of the eye tissue architecture, since better estimates of the ocular tissues optical properties (e.g. reflectivity) are provided. This has been formally demonstrated in the original article by Girard *et al*. [25], and in the following studies [21], [29], [30]. Lastly, although adaptive compensation is useful in all images, it is likely to be more useful when the CSI is poorly visible, e.g. in images where light attenuation is strong (**Figure 1**), emphasizing further the need for compensation for more accurate thickness measurement of the choroid.

Adaptive compensation achieves high intra-grader (ICC: 0.95 to 0.97) and inter-grader (ICC: 0.93 to 0.97) repeatability in CT measurements compared to conventional method (intra-grader ICC: 0.87 to 0.94, inter-grader ICC: 0.90 to 0.93), suggesting the use of adaptive compensation to improve the visualization of CSI and to obtain more reliable CT measurements. However, the high repeatability can be explained by the standardization and strict adherence to a rigorous grading protocol in the present study. In addition, both graders underwent the same training set for standardization purpose before embarking on the actual grading task. The present results are in line in the studies by Ikuno *et al.* [31] and Yamashita *et al.* [32] who reported an inter-grader ICC of 0.97 and 0.94, respectively, for sub-foveal CT measurements. However, in these two previous studies the intra-grader repeatability was not evaluated, and it is not clear how the choroid images, where CSI could not be clearly visualized, were processed.

But the present study has its own limitations, as the measurement of CT was subjective in nature, and was therefore subject to measurement bias. However, our method may be less prone to observer error because the CSI was enhanced by adaptive compensation to give better visibility. An automated, and thus may be more objective, method of measuring CT would be of potential interest to facilitate, speed-up and render operator-independent such analyses.

There are few recent studies on automatic choroidal segmentation in OCT images. Zhang *et al.* proposed an automatic segmentation algorithm for the choroidal vessels using Cirrus OCT in 24 normal subjects. But their aim was to quantify choroidal vasculature thickness and choroicapillaries equivalent thickness rather than the CT [33]. Likewise, Torzicky *et al.* [34] and Duan *et al.* [35] developed the automatic algorithms to detect the boundary between the choroid and sclera based on polarization sensitive OCT which are not commercially available. Although, Tian *et al.* [36] in 2013 proposed an automatic algorithm that could segment choroid in commercially available Spectralis OCT, but their algorithm was tested only on 20 EDI OCT images and need to be validated on more images to prove the robustness of algorithm before its application in clinical studies. While we are preparing the manuscript, a commercially available CT measurement algorithm in swept-source OCT (Topcon Corp., Tokyo, Japan) is being made available. However, in view of lack of supporting agreement studies on the algorithm and manual choroidal segmentation (in both normal and diseased eyes), the accuracy and reliability of automated CT analysis by using swept-source OCT is yet to be established for use in clinical settings.

In conclusion, we described a simplified, semi-automated and practical method “adaptive compensation followed by using Photoshop” that gives excellent intra- and inter-grader reliability (ICC>0.93) to quantify CT in the EDI SD-OCT images. This method has great potential usage in both clinical and population based studies, as reliable and accurate measurements of CT from EDI-OCT images are essential in distinguishing clinically significant change of CT and assisting in risk-profiling for various posterior segment diseases.

## Supporting Information

Appendix S1 **Detailed steps to quantify choroidal thickness using Photoshop.**
(DOCX)
