# Supplementary material for: A Simplified Method to Measure Choroidal Thickness Using Adaptive Compensation in Enhanced Depth Imaging Optical Coherence Tomography
Source: PLoS One. 2014 May 5;9(5):e96661. doi: 10.1371/journal.pone.0096661 (PMC4010516; doi:10.1371/journal.pone.0096661)

**Appendix S1. Detailed steps to quantify choroidal thickness using Photoshop**

**1) Set the Foveal Reference Line**

Firstly we open the image from the file menu, then go the View menu and click on new guides options. In the following window, change the guide orientation to vertical and set the position of the guide and click ok, a reference line is generated (see the screen shots below).


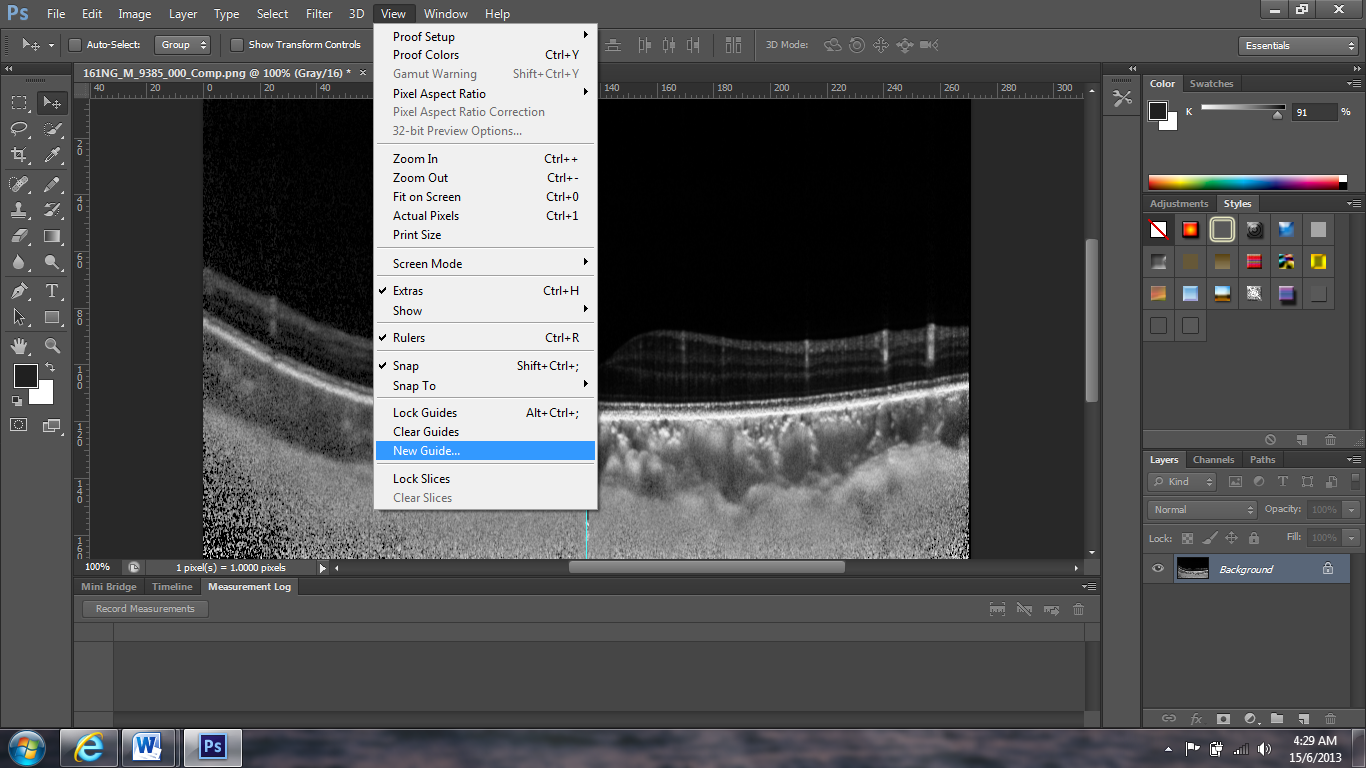


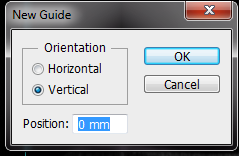


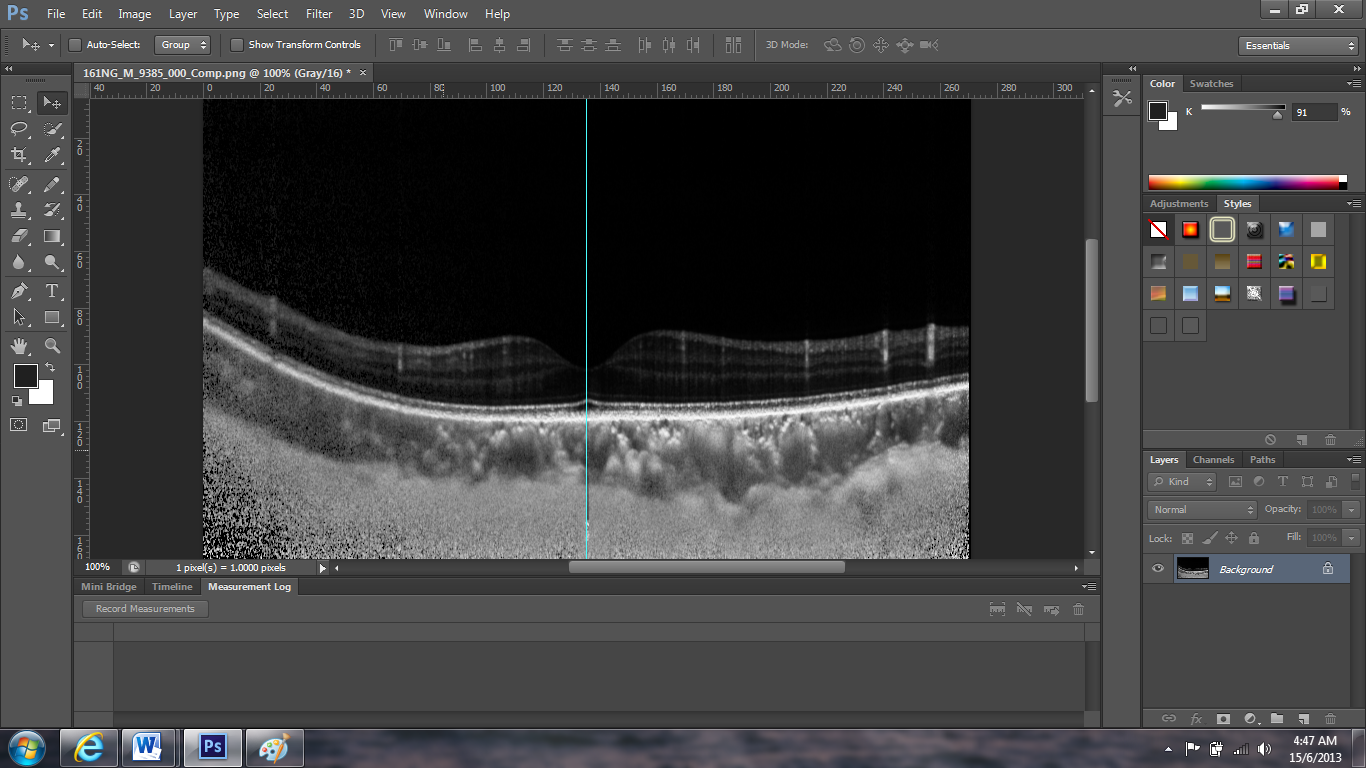


Foveal reference

line

**2) Place the** **Measurement Grid**

We used a grid to indicate accurately our measurement points (sub-fovea, 1.5 and 3 mm nasal and temporal to the fovea) and placed it such that the central point on the grid coincides with the reference line.


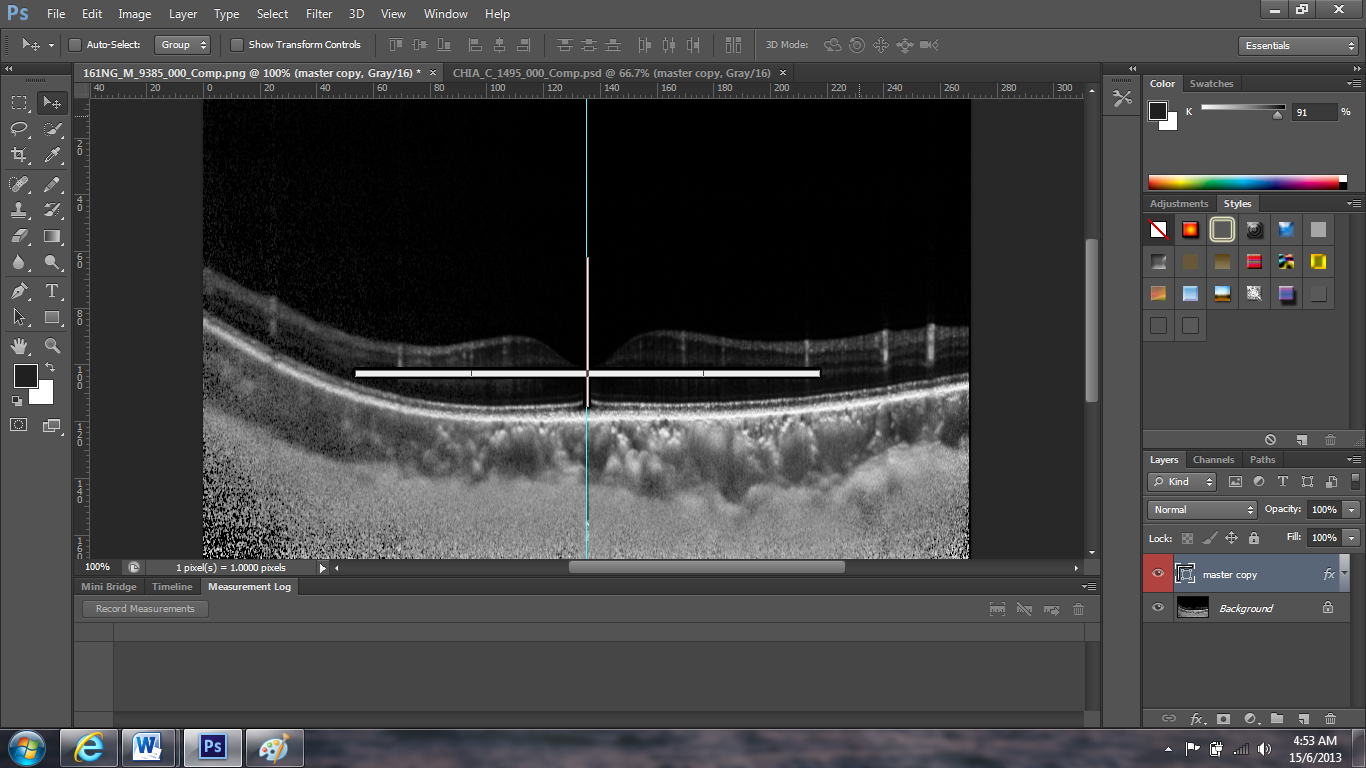


Measurement Grid

**3).** **Set the measurement scale**

Select  *Analysis > Set Measurement Scale > Custom* to open the Measurement Scale dialog box.


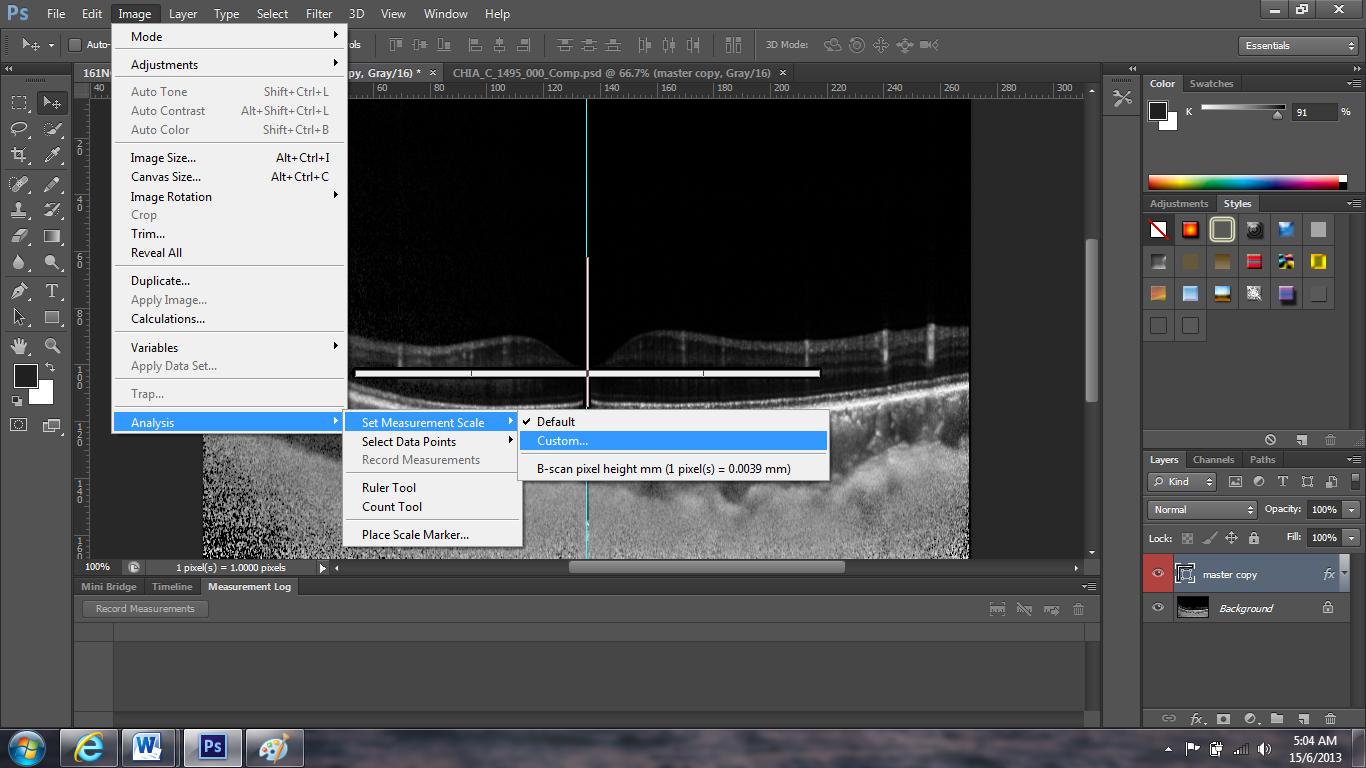


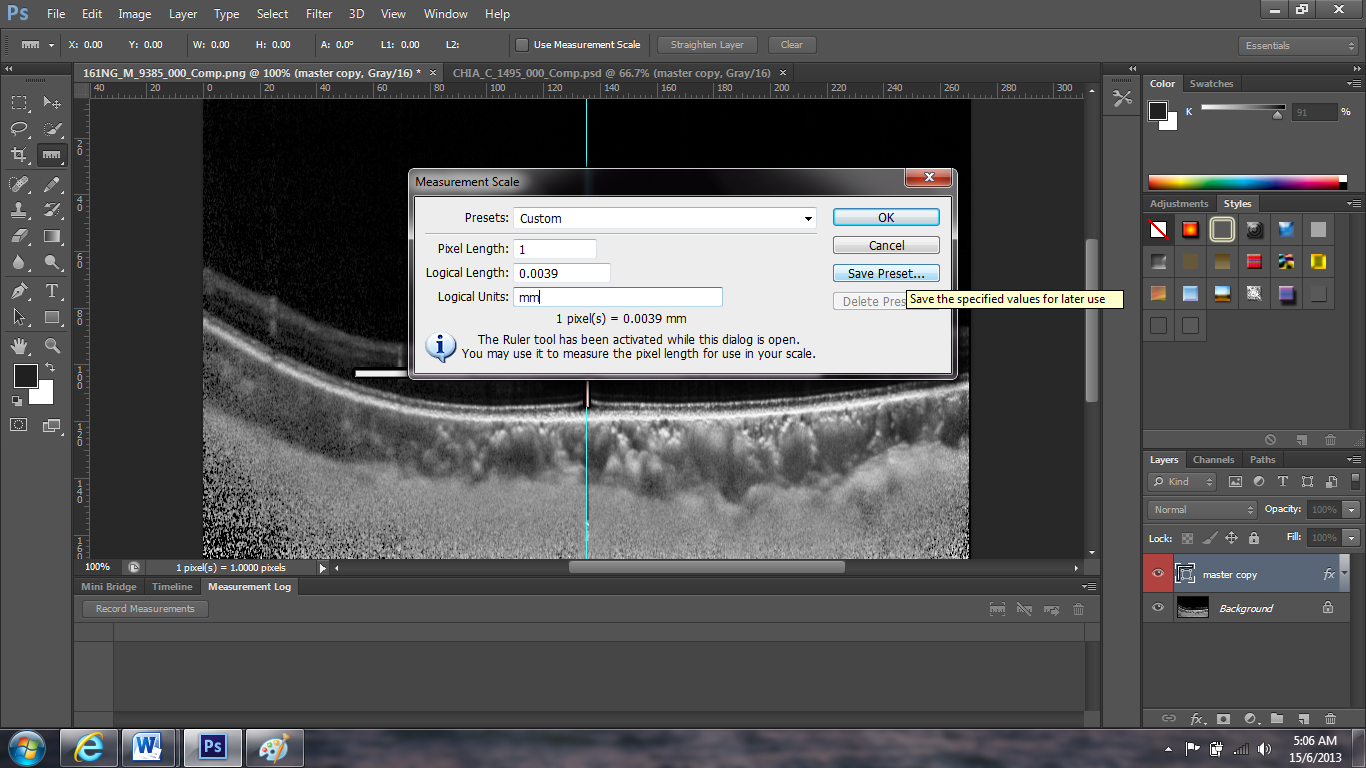


Enter the Logical Length and Logical Units that you want to set equal to the Pixel Length. For example, in our case the Pixel Length is 1, and we want to set a scale of 0.0039 mm per pixel, enter 0.0039 for Logical Length, and millimeter for the Logical Units.

Click OK in the Measurement Scale dialog box to set the measurement scale on the document.

Choose File > Save to save the current measurement scale setting with the document.

**4)** **Perform Measurement**

Choose Analysis> Ruler Tool, or Click the Ruler tool in the toolbox (left hand corner), then use the tool to measure the length of the image.

Click at the starting location for the measuring line and drag to the end location. Release the mouse button to create the measurement line.


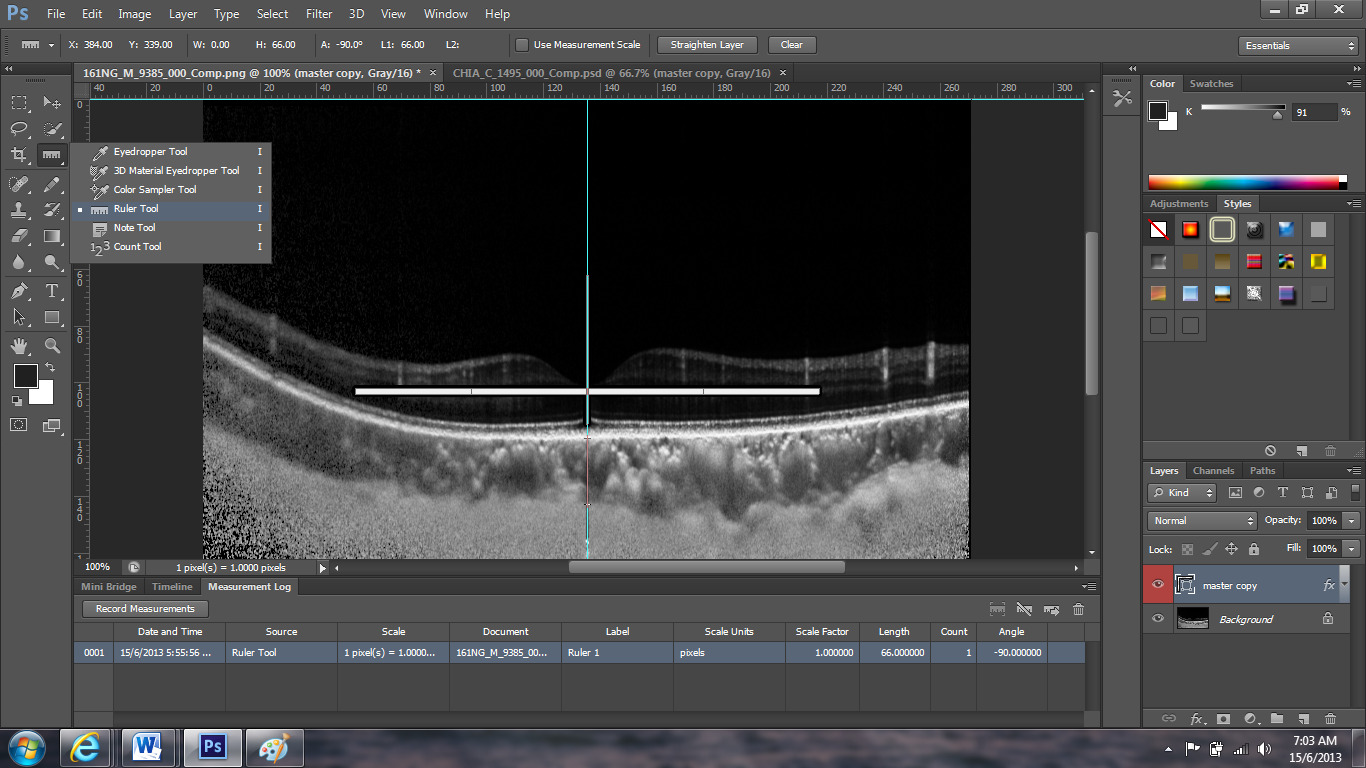


**5) Display and export the Measurement Log** **Data**

Choose Analysis> Record Measurements, or Click Record Measurements in the Measurement Log panel.

Each row in the log represents a measurement set; columns represent the data points in a measurement set. We can reorder columns in the log, sort data in the columns, delete rows or columns, or export data from the log to a comma delimited text file.


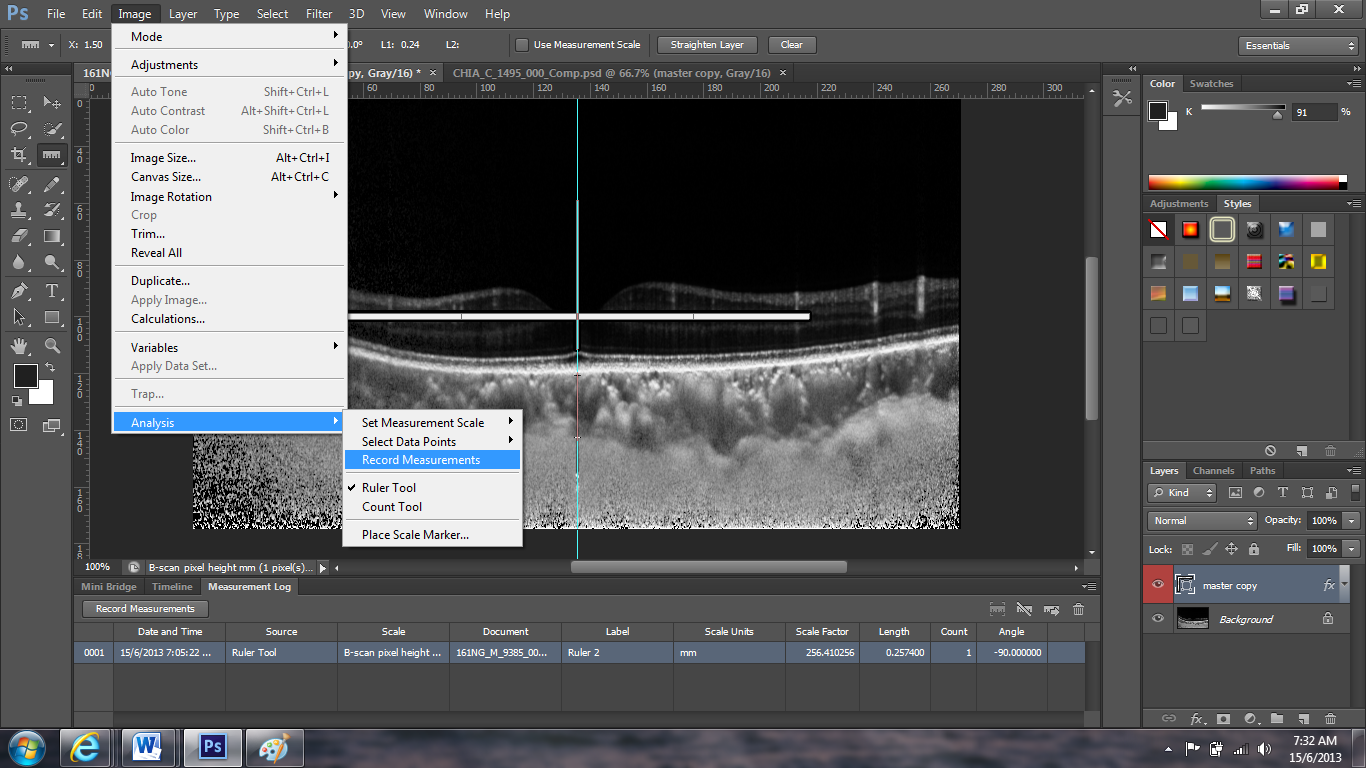


Export data from the Measurement Log into a comma-delimited text file. We can open the text file in a spreadsheet application and perform statistical or analytical calculations from the measurement data.

1. Select one or more rows of data in the log.
2. Choose Export from the Measurement Log options menu or

Click the Export icon at the top of the panel or

Right-click in a row, then select Export from the pop-up menu.

1. Enter a filename and location, and click Save.

The measurements are exported to a comma-delimited, UTF‑8 text file

.


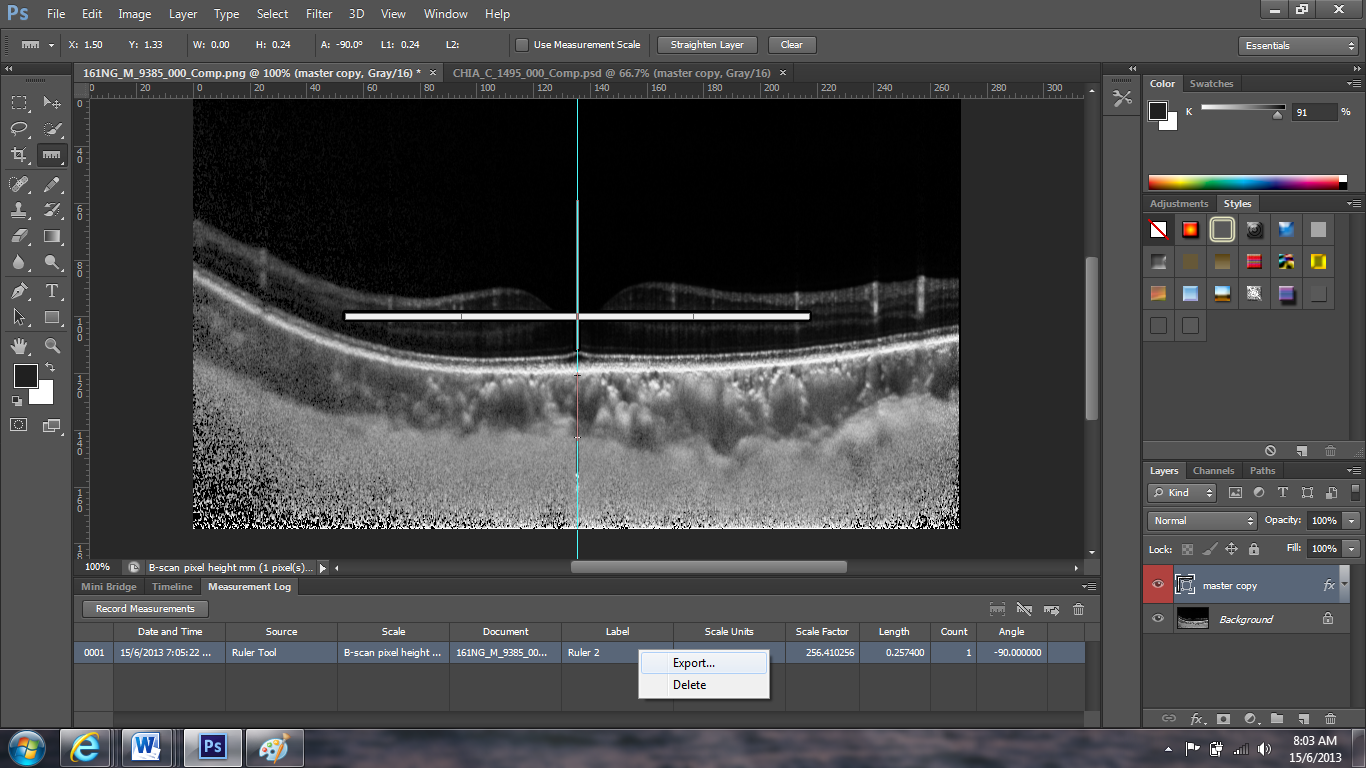

Supplement: Appendix S1 — Detailed steps to quantify choroidal thickness using Photoshop. (DOCX) [file pone.0096661.s001.docx]
